# Bacillus Calmette-Guérin Cases Reported to the National Tuberculosis Surveillance System, United States, 2004–2015

**DOI:** 10.3201/eid2503.180686

**Published:** 2019-03

**Authors:** Zimy Wansaula, Jonathan M. Wortham, Godwin Mindra, Maryam B. Haddad, Jorge L. Salinas, David Ashkin, Sapna B. Morris, Gail B. Grant, Smita Ghosh, Adam J. Langer

**Affiliations:** Centers for Disease Control and Prevention Atlanta, Georgia, USA (Z. Wansaula, J.M. Wortham, G. Mindra, M.B. Haddad, J.L. Salinas, S.B. Morris, G.B. Grant, S. Ghosh, A.J. Langer);; Florida Department of Health, Tallahassee, Florida, USA (D. Ashkin)

**Keywords:** Bacillus Calmette-Guérin, BCG, Mycobacterium bovis BCG, tuberculosis, TB, public health surveillance, tuberculosis and other mycobacteria, United States, bacteria

## Abstract

*Mycobacterium bovis* bacillus Calmette-Guérin (BCG) is used as a vaccine to protect against disseminated tuberculosis (TB) and as a treatment for bladder cancer. We describe characteristics of US TB patients reported to the National Tuberculosis Surveillance System (NTSS) whose disease was attributed to BCG. We identified 118 BCG cases and 91,065 TB cases reported to NTSS during 2004–2015. Most patients with BCG were US-born (86%), older (median age 75 years), and non-Hispanic white (81%). Only 17% of BCG cases had pulmonary involvement, in contrast with 84% of TB cases. Epidemiologic features of BCG cases differed from TB cases. Clinicians can use clinical history to discern probable BCG cases from TB cases, enabling optimal clinical management. Public health agencies can use this information to quickly identify probable BCG cases to avoid inappropriately reporting BCG cases to NTSS or expending resources on unnecessary public health interventions.

## Abstract 2

TOC summary: Clinical history can discern probable BCG cases from TB cases, enabling optimal clinical management.

Countries with a high incidence of tuberculosis (TB) commonly use bacillus Calmette-Guérin (BCG), a live, attenuated strain of *Mycobacterium bovis*, as a vaccine to prevent disseminated tuberculosis disease among children. BCG is also used as first-line treatment for superficial bladder cancer (*1*). Rarely, BCG causes localized infection or disseminated disease with clinical features similar to those caused by *M. tuberculosis*, which causes most TB cases among humans (*2*,*3*). However, the primary diagnostic tests for TB (acid-fast bacillus [AFB] and smear or culture) do not distinguish BCG strains of *M. bovis* from other organisms in the *M. tuberculosis* complex. Nonetheless, the clinical and public health management of the diseases differ. Whereas treatment of TB is always indicated, and symptomatic BCG case-patients might require treatment with anti-TB drugs, asymptomatic BCG case-patients rarely, if ever, need treatment. Unlike typical TB cases caused by *M. tuberculosis*, BCG cases are primarily healthcare-associated, and a traditional TB contact investigation would be unnecessary for BCG cases without pulmonary involvement. For the purposes of this article, we will describe cases caused by non-BCG strains of the *M. tuberculosis* complex as TB disease and cases caused by BCG as BCG disease.

Difficulty distinguishing persons with BCG disease from persons with TB disease also has national surveillance implications. The Council of State and Territorial Epidemiologists (CSTE) collaborates with the Centers for Disease Control and Prevention (CDC) to establish national case definitions for public health notification and reporting purposes (*4*). The laboratory criteria for TB cases, as defined by CSTE, are isolation of *M. tuberculosis* complex from a clinical specimen, demonstration of *M. tuberculosis* complex from a clinical specimen by nucleic acid amplification test, or demonstration of AFB in a clinical specimen if neither culture nor nucleic acid amplification is available. CDC’s Division of Tuberculosis Elimination, part of the National Center for HIV/AIDS, Viral Hepatitis, STD, and TB Prevention, has issued additional guidance stipulating that cases caused by BCG strains, regardless of clinical manifestations, should not be reported as TB cases to the National Tuberculosis Surveillance System (NTSS) (*5*). Difficulty in distinguishing BCG cases from other organisms in the *M. tuberculosis* complex, as well as confusion between the CSTE case definition, which does not specifically exclude positive BCG cultures, and CDC guidance, which does, might explain why some BCG cases are reported as verified TB cases every year. We analyzed characteristics of these BCG cases and the circumstances under which reporting areas decided to report the cases to NTSS.

## Methods

We used genotyping data from the National Tuberculosis Genotyping Service to identify BCG cases reported by the 50 US states and the District of Columbia to NTSS during 2004–2015. We defined a BCG case as a report with a positive laboratory result related to *M. bovis* BCG: spoligotype 676773777777600 and x, y, or z in the second locus of the mycobacterial interspersed repetitive unit–variable-number tandem-repeat analysis (*5*).

By using the standard case report information reported to NTSS, we described the demographic characteristics of persons with BCG. We also compared the clinical characteristics of BCG cases with those of TB cases reported during the same 12-year period.

To clarify the possible clinical spectrum of BCG cases, we more closely examined a convenience sample of locally stored public health records for a subset of 13 such cases reported by Texas and Florida during 2014–2015. We used a standardized medical record abstraction form to collect demographic, clinical, and treatment data for each case. We also conferred with public health practitioners who reported these 13 cases to gather additional information about the clinical manifestations, the subsequent clinical and public health management, and the circumstances under which the public health authority classified these cases as verified TB cases.

Analyses of data collected during routine TB surveillance were determined to be exempt from Institutional Review Board review at CDC. Waivers of consent were obtained in 2 states for the 13 patients who are described in this article because more clinical data were necessary to consider whether these reported BCG cases represented asymptomatic persons with BCG-related laboratory results or persons with symptomatic BCG disease.

## Results

### Surveillance Data

Twenty-six US states reported 118 BCG cases during 2004–2015. In the same period, the 50 US states and the District of Columbia reported 91,065 TB cases. Reporting of BCG cases ranged from a median of 6 reports/year during 2004–2007 to 12 reports/year during 2008–2015.

Of reported BCG cases, 86% occurred among US-born persons. Patients with BCG disease were almost exclusively male (93%) and non-Hispanic white (81%). The patients’ median age was 75 years (interquartile range [IQR] 66–81 years), but 6 cases occurred among children <5 years of age. One of these children was US-born; the others were born in Mexico (n = 2), Ethiopia (n = 1), Japan (n = 1), and Ukraine (n = 1). All of the children had exclusively skeletal sites of disease.

Eighty-seven percent of BCG cases had an extrapulmonary disease site; skeletal and genitourinary sites were most commonly reported (Table 1). Only 20 (17%) of the 118 BCG cases had any pulmonary involvement, including 4 with positive sputum smear results and 3 with cavitary lesions identified through chest radiography. Public health authorities might have considered these BCG cases to be infectious and to warrant contact investigations to prevent transmission of the infection to others, although the attenuated nature of BCG makes it less likely that disease would result even if secondary infections occurred in contacts of the patient. In contrast, most (84%) TB cases involved the lungs.

**Table 1:** Characteristics of BCG-related cases and TB cases reported, National Tuberculosis Surveillance System — United States, 2004–2015*

Characteristic	BCG cases, no. (%)	TB cases, no. (%)
Total cases reported, no. (%)	118 (100)	91,065 (100)
Median age (interquartile range), y	75 (66–81)	47 (31–62)
Age group, y		
0–4	6 (5)	954 (1)
5–14	0	856 (0.9)
15–24	2 (2)	10,398 (11)
25–44	3 (3)	30,236 (33)
45–64	16 (14)	28,327 (31)
>65	91 (77)	20,289 (22)
Sex		
M	110 (93)	56,800 (62)
F	8 (7)	34,265 (38)
White, non-Hispanic	95 (81)	14,330 (16)
US-born	101 (86)	35,215 (38.7)
Pulmonary disease only	15 (13)	67,033 (74)
Extrapulmonary disease only	98 (83)	14,659 (16)
Both pulmonary and extrapulmonary disease	5 (4)	9,336 (10)
Extrapulmonary sites of disease†		
Bones and joints	37 (36)	2,953 (12)
Genitourinary	30 (29)	1435 (6)
Lymphatic system	4 (4)	9,088 (38)
Peritoneal	4 (4)	1,473 (6)
Pleural	3 (3)	5,428 (3)
Meningeal	0	1,203 (5)
Other extrapulmonary sites‡	30 (29)	3,453 (14)
History of long-term care facility residency	11 (9)	1,988 (2)
Diabetes§	15 (21)	7,864 (17)

*BCG, Bacillus Calmette-Guérin; TB, tuberculosis. †Denominators for percentages are based on the preceding 2 rows of all cases with any extrapulmonary involvement (i.e., with or without pulmonary involvement). ‡Include blood, urinary bladder, subcutaneous tissues, and bone marrow. §Diabetes comorbidity data only available starting in 2009; therefore, percentages are based on different denominators (45,786 TB cases and 72 BCG cases).

Social factors and underlying conditions among BCG patients differed from those for TB patients. Diabetes (21% vs. 9%) and residence in a long-term care facility at the time of diagnosis (9% vs. 2%) were more prevalent among BCG patients than TB patients.

### Individual BCG Case Reviews

When we collected additional data for the subset of 13 BCG cases reported by Texas and Florida during 2014–2015, we learned that all 13 had occurred among men with a history of bladder cancer treated with intravesical BCG instillations (i.e., instillation of BCG into the bladder) (Table 2). One of the 13 men was asymptomatic when BCG was isolated from his urine within 6 months of his cancer therapy; he did not require treatment with anti-TB drugs. Two other men were considered to have local infections (i.e., cystitis only) related to their recent BCG instillations. The remaining 10 cases had evidence of BCG dissemination beyond the bladder; we describe 4 of these cases in further detail.

**Table 2:** Clinical characteristics, treatment duration, and outcomes of 13 male patients treated with BCG instillations for bladder cancer and subsequently reported to the National Tuberculosis Surveillance System, 2014–2015*

Patient	Type of complication	Time from BCG instillation into bladder to diagnosis	Site of culture specimen	Duration of treatment	Outcome
1	Asymptomatic	<6 mo	Urine	Not treated	Alive
2	Cystitis	<1 mo	Urine	Not treated	Alive
3	Cystitis	4 mo	Urine	3 weeks	Alive
4	Epididymo-orchitis	ND	Abscess	9 mo	Alive
5	Disseminated	ND	Urine	2 wks	Died
6	Left hip involvement	ND	Abscess	1 mo	Died
7	Parotiditis (case report C)	ND	Abscess	4 mo	Died
8	Prosthetic joint infection (case report B)	9 mo	Synovial fluid	ND	Alive
9	Prosthetic joint infection (case report D)	4 y	Abscess	9 mo	Alive
10	Septicemia	1 y	Blood	ND	ND
11	Septicemia, military (case report A)	<1 mo	Blood	9 mo	Alive
12	Spondylodiscitis	ND	Abscess	1 mo	Died
	Spondylodiscitis	10 y	Abscess	1 mo	Died

*BCG, Bacillus Calmette-Guérin; ND, not documented in the medical and public health records that were available during the case reviews.

### Case Report A

A non–US-born, non-Hispanic white man in his 50s sought care at an emergency department because of fever, hematuria, and dysuria a few days after a traumatic catheterization during BCG instillation for bladder cancer treatment. AFB were visualized on urine microscopic examination, and the patient was prescribed levofloxacin and isoniazid to be taken as an outpatient. Symptoms persisted throughout the following month. Although no abnormal findings were documented on physical examination, AFB were visualized on microscopic examination of a blood specimen. Subsequent chest radiography demonstrated multiple inflammatory nodules (≈15 mm) scattered throughout both lung fields, suggestive of miliary TB. Public health authorities isolated the patient at home, but a contact investigation did not identify any infected contacts. The patient completed 9 months of isoniazid, rifampin, and ethambutol under directly observed therapy. Public health and medical staff confirmed they were aware of the patient’s bladder cancer diagnosis, history of intravesical BCG therapy, and the possibility that the positive *M. tuberculosis* complex culture was BCG-related before genotyping results were available.

### Case Report B

A US-born, non-Hispanic white man in his 80s with a history of bladder cancer sought care from his primary physician because of left hip pain. Five months before the patient’s hip pain began, he had undergone intravesical BCG instillation for treatment of bladder cancer without complications. A month later, he underwent bilateral total hip replacement. *M. bovis* was isolated from culture of synovial fluid from the patient’s left hip joint. A chest radiograph displayed a nodular infiltrate in the right upper lung lobe. At the time of the case review, the patient was receiving isoniazid, rifampin, and ethambutol under directly observed therapy. Public health and medical staff confirmed they were aware of the patient’s bladder cancer diagnosis, history of intravesical BCG, and the possibility that the positive *M. bovis* culture was BCG-related before genotyping results were available.

### Case Report C

A US-born, non-Hispanic white man in his 70s experienced left parotid swelling, weight loss, night sweats, and poor appetite. He had a history of bladder cancer treated with intravesical BCG instillations. Nucleic acid amplification test on the left parotid tissue specimen was positive for *M. tuberculosis* complex, and culture grew *M. tuberculosis* complex. Drug-susceptibility testing indicated resistance to pyrazinamide, which is suggestive of *M. bovis *infection. The patient’s healthcare provider interpreted chest radiography performed at that time as normal. The patient began isoniazid, ethambutol, and rifampin under directly observed therapy; he died 4 months later. Whether or when the healthcare providers suspected BCG as the etiology of the swelling was uncertain.

### Case Report D

A US-born, non-Hispanic white man in his 80s had a history of bladder cancer treated with intravesical BCG for 6 weeks. That same year, he also received a left knee replacement. Four years later, the patient visited his health care provider because of left knee pain of 3 months’ duration. A review of symptoms noted an absence of fever or chills. *M. bovis* was subsequently isolated from culture of a left knee aspirate. The patient received isoniazid, rifampin, and ethambutol under directly observed therapy for 9 months. Public health and medical staff confirmed they were aware of the patient’s bladder cancer diagnosis, history of intravesical BCG, and the possibility that the positive *M. tuberculosis* complex culture was BCG-related before genotyping results were available.

## Discussion

Although CDC advises health departments not to report conditions caused by BCG strains of *M. bovis* to NTSS, 26 states reported 118 BCG cases during 2004–2015. Apart from a few cases occurring among non–US-born young children, BCG cases occurred primarily among US-born older white men. These demographics mirror the primary populations who receive BCG: young children born in TB-endemic countries and older men with bladder cancer (*6*,*7*).

Regardless of age distribution, these patients with BCG typically had extrapulmonary disease, whereas TB patients had pulmonary involvement. This difference probably reflects distinct routes of transmission: *M. bovis* BCG transmission can occur by either intradermal vaccination or intravesical therapy for bladder cancer, whereas *M. tuberculosis* transmission is typically airborne. Non-BCG *M. bovis* typically results from a foodborne transmission route and reportedly has a propensity for extrapulmonary disease (*8*,*9*).

Discerning between actual TB cases and medical complications of BCG therapy is crucial because their clinical and public health management differs. TB treatment is not indicated when BCG is isolated from otherwise asymptomatic persons (*10*,*11*). *M. bovis* BCG acquisition is a rare healthcare-associated event that might require a different type of investigation from the traditional TB contact investigation (*12*). To make that differentiation, clinicians can ask about a history of BCG vaccination or bladder cancer. They can also examine the organism’s drug-susceptibility results; similar to other *M. bovis* strains, BCG is naturally resistant to pyrazinamide. Genotyping results, available through the National Tuberculosis Genotyping Service, can also help health departments identify BCG strains.

Healthcare providers should ask any adult with newly diagnosed TB and a history of bladder cancer detailed questions about previous cancer therapy, even if therapy occurred years earlier. One of the older male patients we describe had been treated for cancer a decade before, and others have reported BCG complications up to 17 years later (*13*). 

Although the 13 cases that we reviewed in detail (Table 2) were a convenience sample, they illustrate a wide spectrum of complications of intravesical BCG therapy for bladder cancer. Other studies have reported a preponderance of disseminated over localized BCG complications (*2*,*14*). In contrast, isolation of BCG from the urine in the absence of clinical signs or symptoms might not require treatment because BCG can be isolated from the urine of asymptomatic patients for up to 16.5 months after completion of the instillation course (*15*). Basing TB treatment decisions on such specimens, without consideration of the clinical picture and patient history, can lead to misdiagnosis and unnecessary treatment.

Numerous published case reports have documented complications induced by BCG vaccination among children that can lead to diverse manifestations, including skeletal complications, although complications rarely occur among immunocompetent children (*16*). Six of the 118 BCG cases we describe here involved children <5 years of age. All had skeletal complications. Most were born in countries where BCG vaccination is routine for all newborns (*17*). Because BCG is not a routine childhood vaccine in the United States, these results support our expectation that vaccination would be the etiology for few BCG cases in NTSS.

This review has certain limitations. NTSS collects limited information about medical conditions other than TB; therefore, we cannot determine whether children with positive BCG cultures had a history of BCG vaccination or whether adults with positive BCG cultures had received treatment for bladder cancer. We also did not know for all 118 BCG cases whether a patient’s BCG therapy had unintentionally led to disseminated disease complications or whether a positive BCG culture was simply incidental (e.g., from the urine of an otherwise asymptomatic person). For this reason, we collected additional data for a subset of 13 reported BCG cases. Although we learned that all of these 13 case-patients had been treated with intravesical BCG, we could not ascertain whether the other 99 adults with BCG disease also had been treated with intravesical BCG for bladder cancer.

Because CDC TB surveillance instructions discourage reporting cases with positive BCG cultures to NTSS and 24 states did not report any BCG cases during 2004–2015, additional BCG cases might have been diagnosed but not reported. Therefore, the case-patients we describe might not represent the epidemiology of BCG disease in the United States. Furthermore, the actual extent of BCG-related complications might be higher.

In conclusion, our review reveals the importance of bladder cancer treatment, rather than BCG vaccination, in the epidemiology of BCG cases in the United States. To avoid misclassification of TB disease, CDC instructions exclude BCG cases from national surveillance for TB. To further clarify that BCG cases should not be reported to NTSS, this exclusion could be explicitly added to the national TB surveillance case definition. Regardless of CSTE case definitions or CDC guidance, adverse events related to BCG or any other medical device or medication should still be reported to the US Food and Drug Administration’s MedWatch (*18*). Nonetheless, distinguishing medical complications of BCG therapy from actual cases of TB disease enables optimal clinical and public health management for individual patients. In addition, early distinction of probable BCG cases from TB cases by public health agencies could prevent unnecessary use of public health resources to respond to these cases.
